# Comparing Media and Law Enforcement Reports on Anti-Asian Hate Incidents During the COVID-19 Pandemic: Data Visualization Approach

**DOI:** 10.2196/70881

**Published:** 2025-09-17

**Authors:** Young Ji Yoon, Su Hyun Shin, Dongwook Kim, Hee Yun Lee

**Affiliations:** 1 Department of Social Work Colorado State University Pueblo Pueblo, CO United States; 2 Department of Family and Consumer Studies The University of Utah Salt Lake City, UT United States; 3 School of Social Work Arizona State University Phoenix, AZ United States; 4 School of Social Work University of Georgia Athens, GA United States

**Keywords:** anti-Asian hate incidents, hate crimes, Asian American and Pacific Islanders, COVID-19, United States

## Abstract

**Background:**

During the COVID-19 pandemic, anti-Asian hate incidents (AAHIs) increased conspicuously. Literature reports discrepancies in how crimes are reported differently in media and law enforcement data, emphasizing potential biases and inconsistencies in AAHI reporting. Understanding the discrepancies in AAHI reporting between the two sources is crucial for improving documentation procedures and addressing systemic issues in reporting mechanisms.

**Objective:**

This study aimed to (1) present the monthly trends in AAHI counts reported by media and law enforcement data from 2020 to 2021, (2) investigate variations in AAHI counts across states and counties for each year, (3) examine discrepancies in AAHI reporting between the two sources at state and county levels during the same period, and (4) delineate differences in the types and geographic distribution of incidents as represented by the two sources.

**Methods:**

This study used two data sources for AAHIs, media data (n=1288) from The Asian American Foundation and law enforcement data (n=1086) from the Federal Bureau of Investigation, for the 2020-2021 period. Descriptive analyses were conducted to evaluate monthly trends, state and county-level variations, and differences in incident types and locations. Ratios of reported incidents between the two sources were calculated to assess discrepancies. Temporal trends were contextualized within key sociopolitical events to offer insights into reporting dynamics.

**Results:**

First, both media and law enforcement data presented a sharp increase in reported AAHIs following the first confirmed COVID-19 case in the United States, peaking around March 2020, coinciding with controversial political rhetoric. A second peak occurred from March to April 2021, immediately following the pandemic’s peak, and was followed by a decline as the situation improved. Second, in the two data sources, the state-level analysis indicated that California, Texas, New York, and Washington consistently reported the highest AAHI counts. In 2021, there were notable increases in reported incidents in states such as Wisconsin and Illinois. County-level data revealed persistent high counts in California, particularly in Los Angeles County. Ratios of AAHI counts between the two data sources presented significant discrepancies, with higher ratios in California and New York. Finally, the analysis of incident types revealed that media data reported a higher proportion of harassment (477/1288, 37%), while the law enforcement data reported more property-related incidents (239/1086, 22%). Regarding location types, media data frequently reported incidents in public areas (515/1288, 40%) and businesses (361/1288, 28%), whereas law enforcement data reported more incidents occurring in residential settings (201/1086, 18.5%).

**Conclusions:**

This study highlighted significant trends and disparities in AAHI reporting between media and law enforcement data, underscoring the need for a nuanced understanding of how these incidents were reported. Practice and policy implications suggested fostering community engagement to support Asian communities while enhancing the accuracy and consistency of hate crime reporting.

## Introduction

### Historical Background of Anti-Asian Hate Incidents

During the public health emergency of the COVID-19 pandemic, the United States has witnessed a significant rise in racially motivated hate incidents involving harassment and physical assault targeting Asian American and Pacific Islanders (AAPIs) [1]. These incidents reflect deeper societal issues of animosity, othering, misinformation, and negative stereotypes toward these minority groups [2-4].

In the late 19th century, Asian immigrants came to the United States for low-cost labor in the mining, agriculture, and garment industries, enduring harsh conditions and being unfairly blamed for disease outbreaks such as tuberculosis and cholera [5-7]. During this period, “yellow peril” fears emerged, centering on the perceived threat to European culture resulting from the expansion of Asian influence, particularly on the West Coast [5]. This led to the enactment of the 1882 Chinese Exclusion Act and the 1924 Immigration Act, which restricted immigration from Asian countries [7,8]. With the onset of World War II and the post-Pearl Harbor attack, Japanese Americans, including citizens and people younger than 18 years of age, were unfairly forced into concentration camps due to suspicions about their allegiance to the Japanese Empire [9]. This historical context underscores the persistent and systemic nature of anti-Asian sentiment in the United States, which has been exacerbated during times of crisis, such as the COVID-19 pandemic.

With the advancements in promoting racial justice and equality in recent decades, AAPIs have become the fastest-growing racial and ethnic group in the United States [10]. However, a considerable number of AAPIs continue to contend with a feeling of not achieving full acceptance in American society, frequently being subject to the perception of being perennial outsiders [11,12]. Another stereotype, the “model minority,” has been applied to AAPIs who have been more successful, partly due to higher educational attainment and income compared to other racial or ethnic minority groups in the United States [13,14].

According to the Othering Theory [15], a racially and socially dominant group tends to distinguish or relegate a nondominant group by designating them as an outgroup, aberrant from the prevailing culture and system [16,17]. When this bias merges with the recognition that the outgroup is implicated in catastrophic historical instances (eg, economic recession and acts of terrorism), the negative sentiments and outward manifestations directed toward the outgroup can be magnified [18]. For example, following the 9/11 attacks, hate crimes witnessed an upsurge, particularly targeting Arab Americans perceived to be affiliated with the group involved in the terrorist attack based on their ethnicity, culture, and religion [19]. Similarly, the COVID-19 pandemic may have provoked the dissemination of blame toward AAPIs, which in turn has contributed to a surge in anti-Asian hate incidents (AAHIs) in the United States.

### A Surge of AAHIs During the COVID-19 Pandemic

Amid the COVID-19 pandemic, the perception of AAPIs as perennial outsiders has been further exacerbated. Between January and March 2020, terms such as “Wuhan virus” or “Chinese virus” were used in news media and by government officials, potentially stigmatizing AAPIs [20-22], particularly those of Chinese or Asian appearance, by associating them with virus transmission. The news media in the United States shed light on the sanitation issues in Wuhan’s wet markets, drawing attention to the consumption of wild animals as a potential cause of the coronavirus [23,24]. While the World Health Organization (WHO) recommended in February 2020 that media organizations use the terms not specifying a certain country or region to indicate the virus, hate crimes targeting AAPIs swelled [25].

In 2019, prior to the pandemic, the Federal Bureau of Investigation (FBI) hate crime data documented 7314 hate crimes, and 55.8% of single-bias incidents stemmed from race, ethnicity, or ancestry [26]. After the outbreak of the pandemic, hate crimes presented about a 13% increase, with 8263 and 8327 incidents reported in 2020 and 2021, respectively, compared to 2019. During the same period, more than 62% of single-bias hate crime offenses were due to race, ethnicity, or ancestry during the same period [25,27]. A survey conducted during the pandemic reported that about 34% of the Asian respondents in the United States experienced bias (32.3% of noncriminal and 19.5% of criminal bias) [2]. Verbal insults were the most common form of noncriminal bias (23.7%), while threats of physical violence were the most common form of criminal bias (13.1%) [2].

The years 2020 and 2021 were marked by the outbreak of COVID-19, public health measures, politicians’ remarks, vaccine rollouts, and prevalence of virus variants [28,29]. Han et al [1] reported on changes in anti-Asian hate crimes by comparing incidents between 2019 and 2020. However, their study covered only a segment of the COVID-19 period, and a research gap persists in studying AAHIs over shorter intervals across an extended period during the pandemic. Given this, it is imperative to investigate the temporal patterning of AAHIs following the outbreak of a pandemic, as this may enable researchers to discern connections between the data and seismic historical events. Such an investigation will reveal how specific pandemic-related events may have influenced the frequency of AAHIs, offering critical insights into the factors that may exacerbate or mitigate these incidents.

### Discrepancies in AAHIs Reported Between Media and Law Enforcement Data

The literature underscored the need for researchers to explore AAHIs, drawing on diverse data sources. According to a study examining police reporting among hate crime victims within the initial 2 months of the pandemic, less than 29% of Asian victims were likely to report their bias experiences to the police, compared to 39%, 41%, and 42% of Hispanic, Black, and White victims, respectively [30]. This suggests that a substantial number of AAHI occurrences do not enter police criminal procedures, indicating the need to explore AAHIs using various data sources to gain insights into multiple phases of these incidents.

For example, Curiel et al [31] compared the frequency of different types of crime reported on Twitter (currently known as “X”) with crime data in several Latin American countries. On social media, about 42.2% and 28.3% of posts were about violent crimes and murder, while only 9.8% and 2.4% were about property and sexual crimes, respectively [31]. Conversely, in official crime data, the majority (65.9%) were about property crimes, while 2.5%, 1.5%, and 0.1% were about sexual crimes, violent crimes, and murder, respectively [31]. The disparity in reported crimes between social media and crime data suggested that media coverage of crimes conveyed fear, caution, or perceptions about crimes that went beyond the incidents themselves.

Recently, several studies have reported empirical evidence of AAHIs during COVID-19 using diverse sources. Lantz and Wenger [2] compared AAHI cases reported by Asians and non-Asians through a web-based survey for the first 2 months of COVID-19. Han et al [1] presented AAHIs reported to police in four major US cities with large Asian populations (New York, San Francisco, Seattle, and Washington, DC) in 2019 and 2020. Prior research on AAHIs throughout the pandemic has covered limited regions or relied on data from surveys [1,2].

Despite these studies, there is a notable lack of effort to examine both mass media and law enforcement data pertaining to the AAHIs. Law enforcement data collects and compiles hate incidents that have been investigated and documented by law enforcement agencies, providing an official record of reported incidents [25]. In contrast, news media data may include a broader range of incidents not reported to law enforcement [32-34]. For example, Chadee and Chadee [35] discovered a disparity in crime reporting between police data and newspapers, using a comparative analysis of the reporting ratios from both sources. Their findings revealed that crimes targeting individuals were overreported in newspapers compared to official police reports, while crimes involving property were underreported [35]. Discrepancies between media and law enforcement data can highlight potential biases in reporting or law enforcement practices [33]. Additionally, comparing types and locations of AAHIs between media and law enforcement data can provide insights into potential discrepancies in reporting, the visibility of incidents in different contexts, and the factors that may influence the occurrence and documentation of these hate incidents in various settings.

### This Study

Based on this groundwork, the purpose of this study is to investigate the disparities in reporting AAHI counts between media and law enforcement data at both state and county levels during the COVID-19 pandemic. By examining these discrepancies, the study aims to elucidate potential biases in reporting practices. Our research is crucial for initiating discussions among scholars and policy makers to address this societal issue by providing compelling evidence. To achieve the study’s aim, we will first illustrate the monthly trends of AAHI counts reported in media and law enforcement data between 2020 and 2021. We will then show the state and county variations in AAHI counts reported each year by both media and law enforcement data, as well as the discrepancies in AAHI count reporting between the two sources at the state and county levels in both years. Additionally, we will investigate the differences in the types and locations of incidents reported in the media and national hate crime data.

Information about safety and exposure to hate crimes is a crucial social determinant of health, shaping behavioral and psychological factors that influence population well-being. While this study does not directly assess these health outcomes, disparities in data sources provide insights that may inform public health discourse and future research on the intersection of crime reporting and community health.

## Methods

### Data and Variables

This study used incident-level data compiled by The Asian American Foundation (TAAF) and the FBI. TAAF collected AAHI data reported in various news media between 2020 and 2021. The data included the media source, date, type, description, location of each incident, and the victim’s gender. TAAF set up and scanned Google News Alerts and Feedly weekly or biweekly to identify AAHI-related news reported in media and blog websites (eg, NextShark) using keywords related to AAPIs and hate incident attributes. Staff members further manually read headlines of articles published in the media and selected those that met the definition of an AAHI case. Although the dataset includes news from AAPI-focused outlets such as AsAmNews and Asian Dawn, only articles published in English were included in the final dataset.

The FBI provided a comprehensive set of incident-level hate crime data, including details on bias types and motivations, criminal acts and types, race or ethnicity, age, and gender of offenders, victims, and arrestees, as well as incident types and locations. The FBI’s data collection process involves two steps. First, a responding officer determines whether an incident is suspected to be bias-motivated. If so, the case is then forwarded to a second-level judgment officer or unit to review, and then reported to the FBI Uniform Crime Reporting program [36]. Although Congress passed the Hate Crime Statistics Act on April 23, 1990, mandating the Attorney General to collect hate crime data from law enforcement at all levels, submitting this data to the FBI Uniform Crime Reporting program remains “voluntary” [36]. Consequently, bias-motivated offenses data might not include all the potential AAHIs if some law enforcement agencies do not participate in the program.

To provide an overview of AAHIs during the COVID-19 period, this study used two variables: (1) AAHI counts and (2) the ratio of AAHIs reported in media data to those in law enforcement data. First, AAHI counts reported in the media were coded as one if a case exhibited bias-motivated acts committed, in whole or in part, because of actual or perceived race or ethnicity, where either: (1) the perpetrator explicitly indicated anti-Asian animus during the act, or (2) contextual evidence strongly suggested that the victim was targeted due to their AAPI identity, as perceived by the AAPI community. Property-related crimes targeting institutions or areas (eg, Chinatown) associated with the AAPI community were also included. Based on these selection criteria, 1288 AAHI cases were collected from media reports. Similarly, in the FBI data, AAHI counts were coded as one based on bias motivation codes assigned to anti-Asian hate intent, resulting in 1086 cases during the same period.

To assess the discrepancy in AAHI counts reported between the two data sources, the study calculated the ratio of AAHI counts reported in the TAAF data to those reported in the FBI data. To avoid having zero values in the denominator, 0.1 was added to both the numerator and the denominator. Thus, the ratio is 1 if both the TAAF and FBI data report 0 AAHI cases. Due to this adjustment, the absolute value of the ratio is not meaningful by itself. However, observing whether the ratio increases or decreases over time or whether it is greater or less than 1 provides valuable insights.

### Ethical Considerations

The dataset used in this study did not include any personal identifiers and, therefore, posed minimal risk to participants. Accordingly, the study was determined to be exempt from the Institutional Review Board review at the researcher’s institution (23-11-7119). The researchers acquired permission to use data collected by TAAF.

### Analysis

For data analyses, this study used STATA (version 18.0; Stata Corp LLC). To illustrate the time trends of AAHI counts from 2020 to 2021, we first aggregated AAHI counts by month and data source. Then, we plot the AAHI counts against each month for each data source. Key events (eg, the first confirmed COVID-19 case in the United States) were also included in the plot to help contextualize and understand the observed patterns.

To illustrate state-level variations in AAHI phenomena, we aggregated AAHI counts by state, year, and data source and then drew heat maps. Additionally, we depicted the ratio of AAHI counts reported between the two sources using heat maps, based on the mean values by state and year. Similarly, county-level analyses were conducted by aggregating AAHI counts by county, year, and data source, followed by creating heat maps. The ratio of AAHI counts reported between the two data sources was also illustrated on heat maps, based on the mean values by county and year.

We then illustrated a bar chart showing the percentage of incident types and incident locations by data source. Due to differences in the terminology used by media sources and FBI data to describe incident types and locations, we standardized the language across both datasets and grouped specific categories into broader ones, as detailed in Tables S1 and S2 in Multimedia Appendix 1. When both the FBI and news media sources reported multiple offense types within a single incident, we coded the first offense type listed as the primary offense, using it to categorize the incident. For example, if an incident was recorded as “Aggravated Assault; Intimidation,” it was categorized under Assault. To ensure consistency and reliability, four researchers collaborated on the coding process and cross-checked each categorization. To examine whether incident types and locations differ between data sources, Pearson chi-square statistics were also calculated to test for statistical differences.

## Results

### Time Trends of AAHI Counts

**Figure 1:**
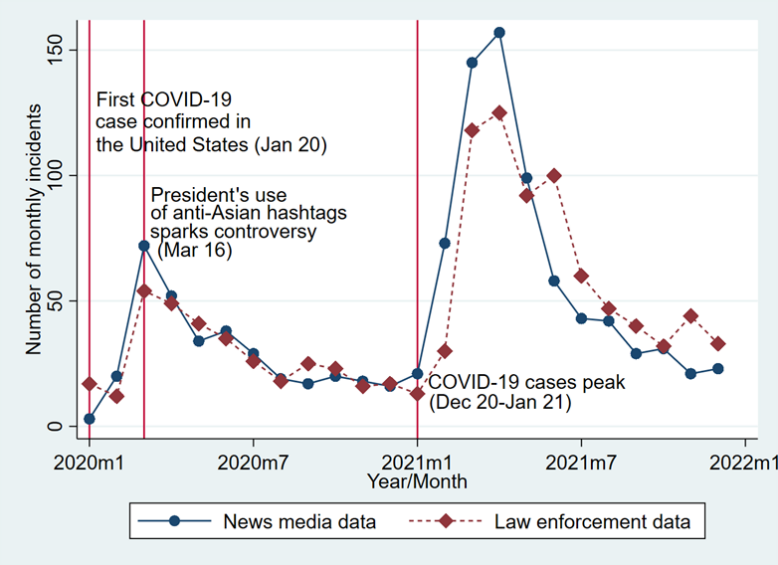
Monthly trends in AAHI counts in the United States, 2020-2021. AAHI: anti-Asian hate incident.

Figure 1 presents the monthly counts of AAHIs reported in both news media and law enforcement data from January 2020 to December 2021. The data points for news media are represented by blue circles connected by solid lines, while law enforcement data are represented by red diamonds connected by dashed lines. Following the first confirmed COVID-19 case in the United States on January 20, 2020, there was a sharp increase in the number of reported incidents in both data sources. The number of incidents peaks around March 2020, coinciding with a politician’s anti-Asian hashtags, immediately following the WHO’s declaration of a global pandemic, which sparked controversy. This initial spike reflects a surge in hate incidents as the pandemic began to take hold and public discourse around its origins intensified.

After the initial spike, there is a gradual decline in the number of reported incidents from April 2020 to October 2020. During this period, both data sources show a similar trend. This decrease suggests a temporary reduction in the intensity or visibility of such incidents following the initial outbreak.

A second peak in reported incidents is observed around March and April 2021, followed by the peak of COVID-19 cases in the United States during December 2020 and January 2021. This spike is more pronounced in the news media data compared to the law enforcement data. The AAHIs’ peak, followed by the pandemic’s peak, suggests that increased stress and fear during this period may have contributed to a resurgence of hate incidents. Following the second peak, both data sources show a noticeable decline in reported incidents throughout 2021. The trends between the two data sources remain relatively aligned, though news media reports generally show higher incident numbers than law enforcement data. This overall decline suggests a reduction in hate incidents as the pandemic situation improved and public attention shifted.

### State Variations of AAHI Counts

**Figure 2:**
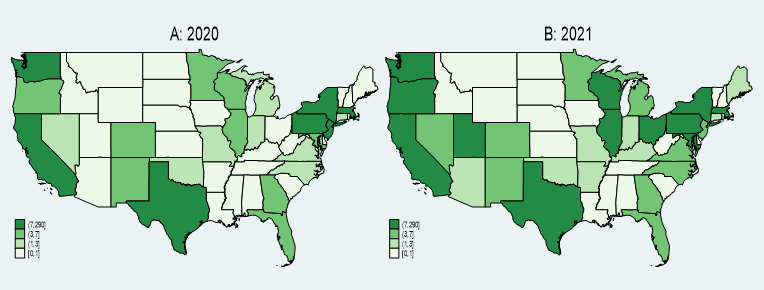
AAHI counts by states, news media data, 2020 and 2021. AAHI: anti-Asian hate incident.

The analysis of AAHI counts reported in news media for the years 2020 and 2021 reveals several key findings (Figure 2). In 2020, California, Texas, New York, and Washington reported the highest numbers of AAHI, indicated by the darkest green shades on the heat map. The central United States exhibited lower reported counts, with many states in lighter green shades. The Northeast and West Coast regions, along with Texas, showed significant concentrations of reported incidents. In 2021, these high counts persisted in California, Texas, New York, and Washington, maintaining their dark green shading. Additionally, increased reported incidents were observed in states such as Wisconsin and Illinois, which displayed darker shades compared to the previous year. The central United States remained predominantly in lighter shades, though some states experienced increases in reported incidents.

**Figure 3:**
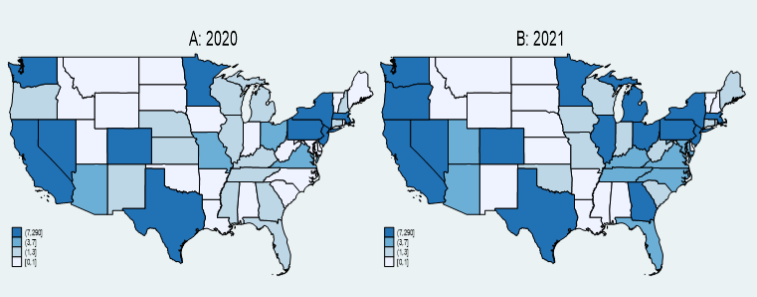
AAHI counts by state, FBI data, 2020 and 2021. AAHI: anti-Asian hate incident; FBI: Federal Bureau of Investigation.

Figure 3 illustrates AAHI counts reported in FBI data across various states for the years 2020 (A) and 2021 (B), respectively. States such as California, Texas, New York, and Washington reported the highest numbers of AAHIs in both 2020 and 2021, as indicated by the darkest blue shades on the maps. This indicates that these states consistently faced significant levels of hate incidents against the AAPIs. In 2021, there was an increase in reported incidents in several states compared to 2020. Notably, states like Tennessee and West Virginia displayed darker shades in 2021, suggesting a rise in reported incidents. The central United States, which predominantly showed lighter shades in 2020, indicating lower counts, saw some states shift to darker shades in 2021. This suggests increased reporting or occurrence of hate incidents in these regions over the year. Parts of the Southeastern United States also exhibited increased reported incidents from 2020 to 2021, with states such as North Carolina becoming more prominent in the data. Along with the consistently high counts in California, Texas, New York, and Washington, the Northeast and parts of the West Coast continued to show significant concentrations of reported incidents in both years.

**Figure 4:**
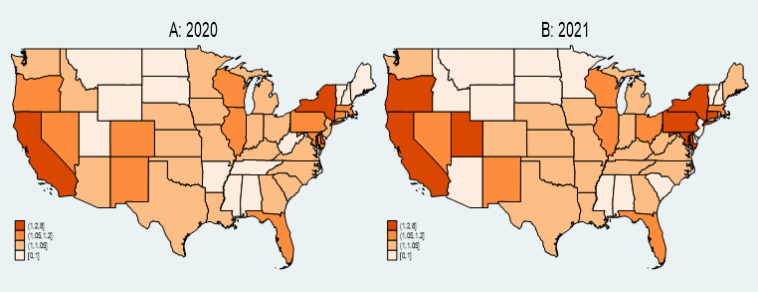
Mean ratio of AAHI counts reported by news media to FBI data by state, 2020 and 2021. AAHI: anti-Asian hate incident; FBI: Federal Bureau of Investigation.

Figure 4 displays the mean ratio of AAHI counts reported between news media and FBI data for 2020 (A) and 2021 (B). The ratio is calculated by dividing monthly county-level AAHI counts reported in news media by those reported in FBI data, with 0.1 added to both the numerator and denominator. We then averaged the ratio by state and by incident year. These heat maps reveal notable trends and disparities between news media and FBI reporting across different states.

In 2020, states such as California, New York, and Maryland exhibited the highest ratios, indicated by the darkest orange shades on the map. This suggests that these states had a significantly higher number of AAHI incidents reported in the news media than the counts reported by the FBI. Conversely, the central United States and parts of the Southeast generally showed lower ratios, represented by lighter orange and beige shades. This indicates a closer alignment between news media and FBI-reported counts or lower overall counts in these regions.

In 2021, the trend of high ratios persisted in states such as California and New York, which continued to display the darkest orange shades. This indicates ongoing significant discrepancies between news media and FBI-reported counts in these states. Additionally, some states, such as Utah, showed an increase in their ratios compared to 2020, indicated by darker shades in the 2021 heat map. This suggests increased news media reporting relative to FBI data in these states. Overall, the central United States and several Southeastern states maintained lower ratios, with lighter shades similar to the previous year, reflecting more consistency between media and FBI-reported counts or lower levels of reporting overall.

### County Variations of AAHI Counts

**Figure 5:**
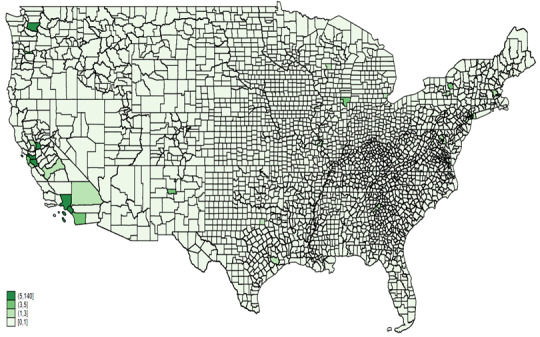
AAHI counts by county from news media data, 2020. AAHI: anti-Asian hate incident.

**Figure 6:**
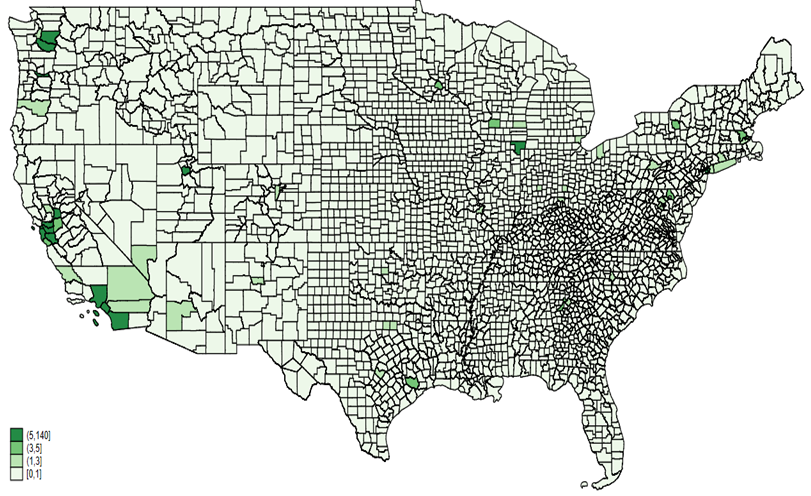
AAHI counts by county from news media data, 2021. AAHI: anti-Asian hate incident.

Figures 5 and 6 provide heat maps illustrating AAHI counts reported in news media across counties for the years 2020 and 2021, respectively. In 2020, the highest counts of AAHI incidents were concentrated in a few key areas, most notably in California, particularly in counties such as Los Angeles. Other notable areas with relatively high counts included some counties in Washington state, New York, and a few scattered regions across the country. However, most counties across the central and southeastern United States reported very few or no incidents, as indicated by the lighter shades or absence of shading on the map.

In 2021, the pattern of high counts in California persisted, with Los Angeles County continuing to show significant numbers of incidents. There was a slight increase in the number of counties reporting incidents across various states, suggesting a broader geographical spread of reported incidents. Washington state and New York again showed notable counts, along with increased reporting in some other states such as Colorado and Illinois. Despite this increase, many counties in the central and southeastern United States still reported lower counts than the coastal and more populated regions.

**Figure 7:**
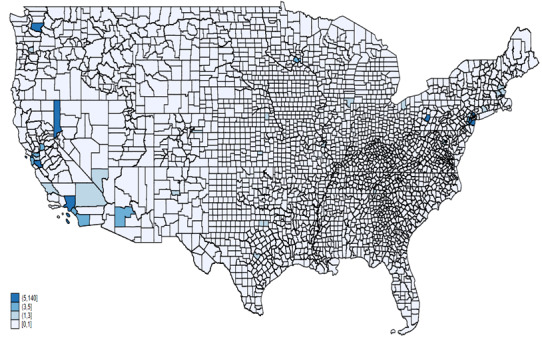
AAHI counts by county from FBI data, 2020. AAHI: anti-Asian hate incident; FBI: Federal Bureau of Investigation.

**Figure 8:**
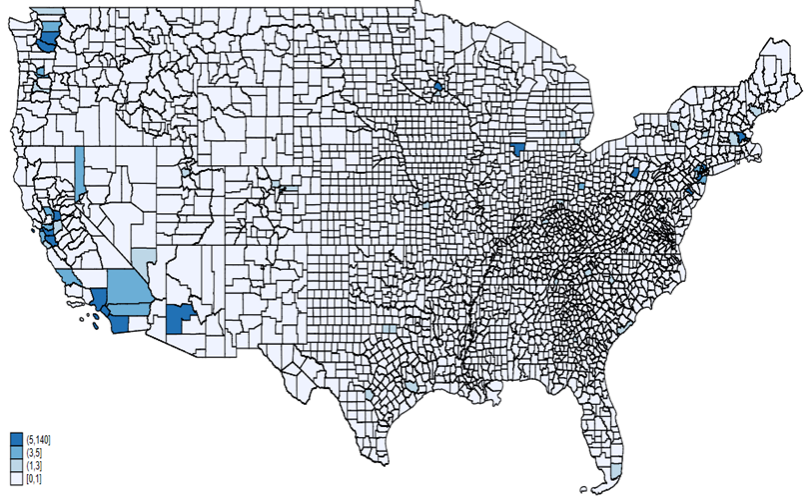
AAHI counts by county from FBI data, 2021. AAHI: anti-Asian hate incident; FBI: Federal Bureau of Investigation.

Figures 7 and 8 illustrate AAHI counts recorded by law enforcement across counties for the years 2020 and 2021, respectively. In 2020, the highest counts of AAHI incidents reported in law enforcement data were concentrated in a few key areas, most notably in California, particularly in counties such as Los Angeles and San Francisco. Other areas with notable counts included counties in Washington state and a few scattered regions in the Northeast, such as New York and Pennsylvania. However, the vast majority of counties across the central and southeastern United States reported very few or no incidents, as indicated by the lighter shades or absence of shading on the map. This suggests that incidents were either less frequent or reported in these regions during 2020.

In 2021, the pattern of high counts in California persisted, with counties like Los Angeles and San Francisco continuing to show significant numbers of incidents. A slight increase in the number of counties reporting incidents across various states suggests a broader geographical spread of reported incidents. Washington state and New York again showed notable counts and increased reporting in some other states, such as Illinois and Minnesota. Despite this increase, many counties in the central and southeastern United States still reported lower counts compared to the coastal and more populous regions, indicating a continued disparity in the geographical distribution of reported incidents.

**Figure 9:**
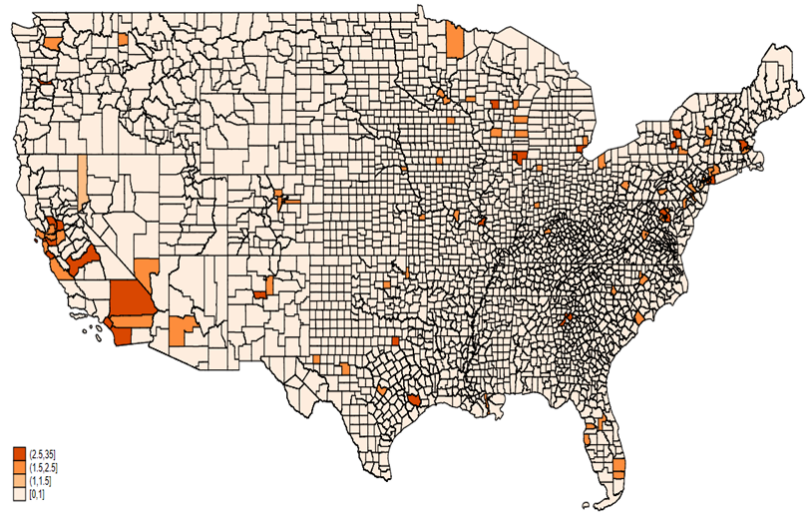
Mean ratio of AAHI counts reported by news media to FBI data by county, 2020. AAHI: anti-Asian hate incident; FBI: Federal Bureau of Investigation.

**Figure 10:**
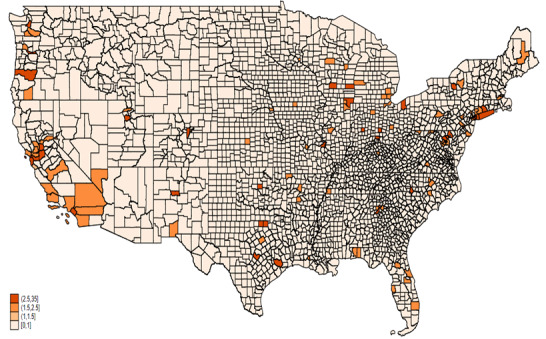
Mean ratio of AAHI counts reported by news media to FBI data by county, 2021. AAHI: anti-Asian hate incident; FBI: Federal Bureau of Investigation.

Figures 9 and 10 show the mean ratio of AAHI counts reported in two data sources by county for 2020 and 2021, respectively. In 2020, the highest ratios, represented by the darkest orange shades, are concentrated in a few areas, particularly in California, including counties such as Los Angeles and San Francisco. This indicates a significantly higher number of incidents reported in the news media compared to FBI data. Other areas with notably high ratios include counties in Washington state, Arizona, and a few scattered regions in the Northeast, such as parts of Pennsylvania and New York. Most counties across the central and southeastern United States show lower ratios, indicated by lighter shades, suggesting more consistency between the two data sources or lower overall reporting levels.

In 2021, the pattern of high ratios persisted in California, with counties like Los Angeles and San Francisco continuing to show significant discrepancies between the news media and FBI data. There was an increase in the number of counties with higher ratios across various states, such as Colorado, Illinois, and parts of the Southeast, indicating a growing disparity in reporting between the two sources in these regions. The central United States still displayed mostly lower ratios, with some increases in counties within Texas and Arizona, suggesting regional variations in the consistency of reporting.

### Incident Types by Data Sources

Table 1 compares the distribution of AAHI types reported in news media versus those reported by the FBI, highlighting differences in the proportions of various incident types between the two sources. In terms of assault incidents, the news media reported that 45% (580/1288) of incidents were assaults, while the FBI reported a slightly higher proportion at 47% (510/1086). This indicates that both sources identify assault as the most common type of incident, with nearly identical proportions. For harassment incidents, there is a noticeable difference between the two sources. The news media reported that 37% (477/1288) of incidents were classified as harassment, whereas the FBI reported a lower proportion of 30% (326/1086). This suggests that the news media reports a higher proportion of harassment incidents than the FBI. For property-related incidents, the FBI reported a significantly higher proportion at 22% (239/1086), compared to just 17% (219/1288) reported by the news media. This indicates that the FBI data includes many more property-related incidents than the news media reports. When we conducted Pearson chi-squares analysis, the value was 32.9072, which is significant at *P*<.001. This suggests that the types of incidents reported statistically differ between the two data sources.

**Table 1:** Percentage of incident types by data sources.

	News media (n=1288), n (%)	Law enforcement (n=1086), n (%)
Assault	580 (45)	510 (47)
Harassment	477 (37)	326 (30)
Property-related	219 (17)	239 (22)
Shunning	13 (1)	0 (0)
Other	0 (0)	11 (1)

### Incident Location Types by Data Sources

Table 2 compares the distribution of AAHIs by location type as reported in news media versus those reported by the FBI, highlighting differences in the proportions of incidents occurring in various settings between the two sources. In the news media reports, the most common location for AAHI incidents was public areas, accounting for 40% (515/1288) of incidents. This is significantly higher than the proportion reported by the FBI, where 33% (358/1086) of incidents occurred in public areas. This discrepancy suggests that news media reports might focus more on incidents occurring in public settings than FBI reports. Businesses were the second most common location for AAHI incidents in news media, with 28% (361/1288) of incidents reported in this setting. In contrast, the FBI reported a slightly lower proportion of 22% (239/1086) for incidents occurring in businesses. Both sources highlight businesses as a major location for AAHI incidents, though the proportions differ. A notable discrepancy is observed in transportation settings, where 13% (167/1288) of incidents were reported in the news media compared to just 2% (22/1086) in FBI data. In contrast, incidents in home or housing settings accounted for 9% (116/1288) of news media reports but 18.5% (201/1086) of FBI reports. Schools were another notable location, but both sources had smaller proportions. The news media reported 3% (39/1288) of incidents occurring in schools, while the FBI reported a slightly lower proportion of 1% (11/1086). Similarly, the news media reported 3% (39/1288) of incidents occurring in colleges or universities, compared to 1% (11/1086) in the FBI data. This indicates that incidents in educational settings are less frequently reported compared to public areas and businesses. Additionally, health care settings and hospitals or clinics were reported more frequently by the FBI (22/1086, 2%) compared to the news media (8/1288, 0.6%). The Pearson chi-square statistic is 214.1602, significant at *P*<.001, implying that AAHI incident location types differ significantly between the two data sources.

**Table 2:** Percentage of incident locations by data sources.

Locations	News media (n=1288), n (%)	Law enforcement (n=1086), n (%)
Public area	515 (40)	358 (33)
Business	361 (28)	239 (22)
Transportation	167 (13)	22 (2)
Home or housing	116 (9)	201 (18.5)
College or university	39 (3)	11(1)
School	39 (3)	11 (1)
Place of worship	21 (1.6)	7 (0.6)
Health care setting	8 (0.6)	22 (2)
Online	5 (0.4)	11 (1)
Other	10 (0.8)	195 (18)

## Discussion

### Principal Findings

Our result demonstrates significant fluctuations in the number of reported AAHIs over the course of the COVID-19 pandemic. Key triggers such as the initial outbreak, controversial political statements, and peaks in COVID-19 cases appear to correlate with spikes in reported incidents. Both news media and law enforcement data exhibit similar overall trends. After May 2021, the crossover point in this sample, AAHIs reported in law enforcement data were modestly higher than those in media reports. This shift may be attributed to the Centers for Disease Control and Prevention’s announcement that fully vaccinated individuals in the United States no longer needed to wear masks, which potentially led to increased social interactions. Consequently, media coverage of AAHIs might have shifted focus or decreased due to changing public priorities.

The relationship between spikes in reported incidents and key events such as the initial outbreak of COVID-19, controversial political statements, and peaks in COVID-19 cases underscores the influence of societal and political factors in driving hate incidents. This evidence is congruent with previous literature indicating that when prejudice against a minority group is compounded by the recognition of the outgroup’s association with significant historical events, negative sentiments and expressions directed toward the outgroup can be intensified [18]. Additionally, our findings suggest that public messaging and political rhetoric may significantly influence the occurrence and reporting of AAHIs. Previous literature indicated that politicians’ narratives to tighten the border and curb immigration had increased violence toward specific racial, ethnic, or spiritual communities [37]. Similarly, anti-Muslim tweets from a prominent political figure have been shown to increase xenophobic tweets by his followers, the number of cable news mentions of Muslims, and hate crimes targeting this population [38]. Content analysis has demonstrated a significant increase in tweets blaming Chinese and other Asians for the virus, reinforcing the Yellow Peril and perpetual foreigner stereotypes, following the use of phrases such as “Chinese virus” or “Kung flu” in a prominent politician’s remarks [22]. While our analysis is descriptive, it provides valuable context that sounds the alarm for politicians and government officials to recognize the impact of their rhetoric, which can shape public opinion and incite further hostility and violence, such as hate incidents.

In both media and FBI data, states such as California, Texas, New York, and Washington consistently reported the highest numbers of incidents, highlighting lingering challenges in these regions. County-level analyses indicate that both media and FBI data consistently reported the highest numbers of AAHI incidents in Los Angeles and San Francisco counties for both 2020 and 2021. This indicates a significant overlap between media and law enforcement reporting in these areas. The high number of AAHI incidents can be attributed to the significant Asian populations in these regions. In 2019, California had the largest Asian population in the nation, with approximately 6.7 million people, followed by New York (1.9 million), Texas (1.6 million), New Jersey (958,000), and Washington (852,000) [39]. Together, these five states were home to 55% of the Asian population in the United States. Community organizations can leverage these findings to advocate for more resources and support services for AAPI communities in these high-incident areas [40]. This includes mental health services, legal assistance, and community safety programs. The consistent identification of these high-incident areas enables targeted interventions by policy makers, community leaders, and law enforcement, allowing for a more effective allocation of resources to prevent and address AAHI.

Both media and FBI data showed an increase in the geographical spread of reported incidents in 2021 compared to 2020. However, there are discrepancies in the overall number of incidents reported. Media data tend to show a wider distribution of lower-count incidents across more counties, suggesting more extensive coverage or awareness. In contrast, FBI data show fewer counties with reported incidents. Additionally, both datasets consistently show lower counts in the central and southeastern United States, with fewer reported incidents in these regions. These findings highlight the need for further research to explore the underlying reasons for discrepancies in reporting between media and law enforcement. It is essential to examine whether lower counts in certain regions reflect actual prevalence or simply lower reporting practices. Understanding these factors can help develop plans to bridge the gap and ensure comprehensive and full documentation of AAHI.

The authors uncovered notable differences in how incident types are reported by news media versus the FBI. While both sources agree on the high prevalence of assault incidents, the news media reports a substantially higher proportion of harassment. In contrast, the FBI data includes a much higher proportion of property-related incidents. Our results align with previous findings showing that law enforcement data have a higher proportion of property-related crime reporting compared to social or news media data [31,35]. News media may prioritize and highlight incidents that are more sensational or resonate more with public interest through editorial decisions, including those involving harassment. In contrast, property-related incidents, such as vandalism or property damage, are more concrete to document and classify under hate crime statutes. Because the losses are more visible and tangible, individuals are more likely to report these incidents to law enforcement. Additionally, Asian Americans tend to underreport their hate incident experiences to police because they may believe that the damage is minor, even when it is not, or they are unsure if they have been affected [30]. As a result, minor incidents, categorized as harassment, are likely underreported to law enforcement due to these two major reasons.

Our results reveal significant differences in the reported locations of AAHI incidents between news media and FBI data. While both sources identify public areas and businesses as common locations, the news media emphasizes public area incidents more heavily, whereas the FBI data highlights incidents in residential settings and others. News media may prioritize incidents that occur in public areas because these incidents are more likely to capture public attention and provoke strong emotional reactions. Public area incidents often involve multiple witnesses and can be more triggering, making them more newsworthy. In contrast, individuals may be more likely to report incidents that occur in residential settings to law enforcement due to the personal and private nature of these incidents. Property damage, home invasions, or harassment at home are often perceived as serious threats to personal safety, leading to formal police reports.

In sum, our descriptive analyses reveal a discrepancy in AAHI reporting between the two sources, with the FBI data showing fewer incidents compared to media data. A growing body of research emphasizes the obstacles that hinder the reporting and processing of hate crimes [32,34,41]. A study identified reasons for not reporting violent bias experiences to police, including perceptions that reports were not important to police, that they were personal matters, that police were inefficient, that police were biased, that it was not clear it was a crime, and that individuals were advised not to report [42]. Another study described that the underreporting of crimes was related to fear of consequences, a lack of trust in law enforcement agencies, and previous police discrimination experiences [32,34]. Tessler et al [41] posited that recent Asian immigrants may not fully grasp and navigate the legal system or know how to report crimes, especially those charged as hate crimes. Studies also suggest that Asian Americans might engage in social withdrawal as a defensive strategy driven by fear, leading to a self-imposed limitation on their movements and a reluctance to report instances of hate crime experiences [2,30]. Additionally, this underreporting may be attributed to the varying definitions and legal frameworks for hate crimes across different states. A study reported that states with more comprehensive definitions of hate crimes report a greater incidence of hate incidents [43]. This underscores the need for more comprehensive and consistent hate crime reporting and classification to better understand and address hate-fueled attacks.

### Practice and Policy Implications

Our findings underscore the impact of societal and political influences on AAHIs and their reporting in both media and law enforcement data, leading to the following practice and policy implications. First, practitioners and policy makers should prioritize building stronger relationships with AAPI communities to encourage the reporting of hate crimes. This can be achieved by fostering trust through community outreach programs, providing organized and detailed information on how to report incidents, and ensuring support systems are in place for those affected. Addressing barriers to reporting, such as fear of retaliation or distrust in law enforcement, is essential for increasing reporting rates. Additionally, given the observed discrepancies between media and law enforcement reports, it is crucial to enhance mechanisms for reporting and tracking AAHIs. This includes developing standardized criteria for reporting and classifying hate crimes across jurisdictions to improve consistency and accuracy. Implementing a consistent and organized reporting system, along with educating law enforcement personnel about this system, could help reconcile discrepancies between media and law enforcement data and ensure comprehensive documentation of incidents.

From a public health standpoint, our findings contribute to building awareness of AAHIs, which can inform and guide public health professionals in addressing their mental and physical health consequences. Racially motivated hate not only harms those affected but also disrupts the social fabric, affecting their families, friends, and broader community. Understanding how hate crimes and incidents are identified and framed is a crucial step toward building health professionals’ capacity to recognize trauma associated with hate crimes and provide culturally competent care. Additionally, public health campaigns can leverage this awareness to foster solidarity, encourage community-based reporting, and promote a sustained response to mitigate the impact of hate crimes. At the policy level, it is vital to integrate anti-hate crime initiatives into broader public health strategies. Policy makers should advocate for laws that provide adequate protection for AAPI individuals and communities, ensuring resources for mental health care and community healing.

### Limitations

This study has several limitations that should be acknowledged. First, the reliance on two primary data sources, media reports compiled by TAAF and law enforcement data provided by the FBI, may introduce inherent biases. Media reports could overrepresent sensational or newsworthy incidents, while law enforcement data might underrepresent incidents due to underreporting by individuals affected or inconsistencies in hate crime classification across jurisdictions. The use of open-source media data without standardized criteria for article selection may result in observation bias, potentially leading to an incomplete representation of relevant incidents. Moreover, online hate incidents, which are increasingly significant, fall outside the scope of this study. Second, the study’s descriptive analysis does not account for potential confounding variables, such as pandemic-related countermeasures (eg, lockdowns and vaccine rollout) that could influence the observed trends and discrepancies in AAHI reporting. Variations in state laws, differences in local media coverage, and the varying capacity of law enforcement agencies to investigate and report hate crimes were not controlled for, which could contribute to discrepancies between the data sources. Third, the study focuses solely on reported incidents, missing the experiences of AAPIs who may have faced discrimination or hate incidents but did not report them. This limitation suggests that the actual prevalence of AAHIs could be higher than what is documented. Additionally, excluding social media data may have resulted in an incomplete picture of the AAHI landscape during the pandemic. Furthermore, although the dataset includes English-language content from AAPI-focused outlets such as AsAmNews and Asian Dawn, non-English articles were excluded. This may lead to underrepresentation of incidents covered only in other languages. Finally, the generalizability of the findings is limited by the focus on the COVID-19 pandemic period. While the study provides valuable insights into AAHI trends during this specific timeframe, the findings may not fully apply to other periods or the broader context of anti-Asian sentiment outside of a pandemic setting. Future research should address these limitations by incorporating more data sources, controlling for additional variables, and extending the analysis to other periods and social contexts.

### Conclusions

This study provides critical insights into the reporting discrepancies and trends of AAHIs during the COVID-19 pandemic, highlighting the complex interplay between societal, political, and media influences on the documentation of hate crimes. Our findings reveal significant fluctuations in reported AAHIs corresponding with key events, such as the onset of the pandemic, peaks in COVID-19 cases, and politically charged rhetoric, underscoring the impact of public discourse on the prevalence and visibility of hate crimes. The observed discrepancies between media reports and law enforcement data, particularly in incident types and locations, suggest that the current methods of documenting and reporting these incidents are insufficient and may fail to capture the full scope of the issue.

Moreover, the geographical analysis indicates persistent high counts of AAHIs in states and counties with significant AAPI populations, emphasizing the need for targeted interventions in these areas. However, the disparities between media and law enforcement data across different regions and the underreporting of incidents in certain states and counties underscore the necessity for a more standardized and comprehensive approach to hate crime documentation. Such an approach would improve the accuracy and reliability of data, enabling policy makers and community leaders to develop more effective strategies to combat hate crimes.

Addressing the discrepancies in AAHI reporting is paramount for advancing our understanding of hate crimes and fostering a safer environment for AAPI communities. Future research should focus on enhancing the consistency of hate crime data collection across various sources, while also exploring the underlying reasons for underreporting and regional disparities. By implementing standardized reporting systems and fostering stronger community-law enforcement relationships, we can work toward a more accurate representation of hate crimes, ultimately contributing to the development of informed and effective policies aimed at eradicating anti-Asian hate and promoting social justice.

## Appendix

Multimedia Appendix 1 The categorization of incident types and locations by data sources.

## Abbreviations

AAHI: anti-Asian hate incident
AAPI: Asian American and Pacific Islander
FBI: Federal Bureau of Investigation
TAAF: The Asian American Foundation
WHO: World Health Organization

## Data Availability

The datasets used in this study are publicly available in the FBI UCR repository [45] and the TAAF repository [46].

## References

[1] Han S, Riddell JR, Piquero AR (2023). Anti-Asian American hate crimes spike during the early stages of the COVID-19 pandemic. J Interpers Violence.

[2] Lantz B, Wenger MR (2023). Anti-Asian xenophobia, hate crime victimization, and fear of victimization during the COVID-19 pandemic. J Interpers Violence.

[3] Roberto KJ, Johnson AF, Rauhaus BM (2020). Stigmatization and prejudice during the COVID-19 pandemic. Adm Theory Prax.

[4] Vidgen B, Botelho A, Broniatowski D, Guest E, Hall M, Margetts H (2020). Detecting East Asian prejudice on social media. ArXiv. Preprint posted online on May 8, 2020.

[5] Lee E (2007). The “Yellow Peril” and Asian exclusion in the Americas. Pac Hist Rev.

[6] Lui MT (2005). The Chinatown Trunk Mystery: Murder, Miscegenation, and Other Dangerous Encounters in Turn-of-the-Century.

[7] Chinese immigration and the Chinese Exclusion Acts. Office of the Historian, Foreign Service Institute.

[8] Stanciu C (2020). Native acts, immigrant acts: citizenship, naturalization, and the performance of civic identity during the progressive era. J Gilded Age Prog Era.

[9] Luther CA (2019). Reflections of cultural identities in conflict. Journalism Hist.

[10] (2021). Key facts about Asian Americans, a diverse and growing population. Pew Research Center.

[11] Cheryan S, Monin B (2005). "Where are you really from?": Asian Americans and identity denial. J Pers Soc Psychol.

[12] Li Y, Nicholson HL (2021). When "model minorities" become "yellow peril"—othering and the racialization of Asian Americans in the COVID-19 pandemic. Sociol Compass.

[13] Lee A, Weiss G, Murphy AV, Salamon G (2019). Model minority. 50 Concepts for a Critical Phenomenology.

[14] (2012). The rise of Asian Americans. Pew Research Center.

[15] Weis L (1995). Identity formation and the processes of "Othering": unraveling sexual threads. J Educ Found.

[16] Gover AR, Harper SB, Langton L (2020). Anti-Asian hate crime during the COVID-19 pandemic: exploring the reproduction of inequality. Am J Crim Justice.

[17] Jefferson T (2015). What is racism? Othering, prejudice and hate-motivated violence. Int J Crime Justice Soc Democr.

[18] Byers BD, Jones JA (2007). The impact of the terrorist attacks of 9/11 on anti-islamic hate crime. J Ethn Crim Justice.

[19] Disha I, Cavendish JC, King RD (2011). Historical events and spaces of hate: hate crimes against Arabs and Muslims in post-9/11 America. Soc Probl.

[20] John T, Guy J (2020). A visual guide to the Wuhan coronavirus. CNN.

[21] Palmer E (2020). 'Chinese coronavirus' trends as Kevin McCarthy accused of being racist over COVID-19 remarks. Newsweek.

[22] Zheng J, Zompetti JP (2023). ‘I’m not a virus’: Asian hate in Donald Trump’s rhetoric. Asian J Commun.

[23] Gomera M How to prevent outbreaks of zoonotic diseases like COVID-19. Aljazeera.

[24] Mackenzie JS, Smith D (2020). COVID-19: a novel zoonotic disease caused by a coronavirus from China: what we know and what we don't. Microbiol Aust.

[25] (2021). Hate crime statistics. Federal Bureau of Investigation.

[26] (2021). Hate crime statistics, 2019. Federal Bureau of Investigation.

[27] (2022). Hate crime statistics, 2020. Federal Bureau of Investigation.

[28] (2021). Federal emergency authorities. MACPAC.

[29] McNamara LA, Wiegand RE, Burke RM, Sharma AJ, Sheppard M, Adjemian J, Ahmad FB, Anderson RN, Barbour KE, Binder AM, Dasgupta S, Dee DL, Jones ES, Kriss JL, Lyons BC, McMorrow M, Payne DC, Reses HE, Rodgers LE, Walker D, Verani JR, Schrag SJ (2022). Estimating the early impact of the US COVID-19 vaccination programme on COVID-19 cases, emergency department visits, hospital admissions, and deaths among adults aged 65 years and older: an ecological analysis of national surveillance data. Lancet.

[30] Lantz B, Wenger MR (2021). Are Asian victims less likely to report hate crime victimization to the police? Implications for research and policy in the wake of the COVID-19 pandemic. Crime Delinq.

[31] Curiel RP, Cresci S, Muntean CI, Bishop SR (2020). Crime and its fear in social media. Palgrave Commun.

[32] Erentzen C, Schuller R (2020). Exploring the dark figure of hate: experiences with police bias and the under-reporting of hate crime. Can J Criminol Crim Justice.

[33] Feldman JM, Gruskin S, Coull BA, Krieger N (2017). Correction: quantifying underreporting of law-enforcement-related deaths in United States vital statistics and news-media-based data sources: a capture-recapture analysis. PLoS Med.

[34] Vergani M, Navarro C (2021). Hate crime reporting: the relationship between types of barriers and perceived severity. Eur J Crim Policy Res.

[35] Chadee D, Chadee M, Weiss G, Murphy AV, Salamon G (2015). Media and fear of crime: an integrative model. Psychology of Fear, Crime and the Media.

[36] Global Law Enforcement Support Section (GLESS), Crime and Law Enforcement Statistics Unit (CLESU) (2022). Hate crime data collection guidelines and training manual. Criminal Justice Information Services (CJIS) Division Uniform Crime Reporting (UCR) Program.

[37] Hodwitz O, Massingale K (2021). Rhetoric and hate crimes: examining the public response to the Trump narrative. Behav Sci Terror Polit Aggress.

[38] Müller K, Schwarz C (2023). From hashtag to hate crime: twitter and antiminority sentiment. Am Econ J Appl Econ.

[39] Budiman A, Ruiz NG (2021). Key facts about Asian Americans, a diverse and growing population. Pew Research Center.

[40] Francisco C, Cruz M, Phuong A, Jeung R, Yoo G (2022). Healing in community and responding with leadership: addressing the pandemic and anti-Asian hate through community service learning. AAPI Nexus Policy Pract Community.

[41] Tessler H, Choi M, Kao G (2020). The anxiety of being Asian American: hate crimes and negative biases during the COVID-19 pandemic. Am J Crim Justice.

[42] Pezzella FS, Fetzer MD, Keller T (2019). The dark figure of hate crime underreporting. Am Behav Sci.

[43] Stacey M (2015). The effect of law on hate crime reporting: the case of racial and ethnic violence. Am J Crim Just.

[44] OpenAI (2023). ChatGPT-4. Large language model.

[45] Federal Bureau of Investigation The Hate Crime Master File, 2020 and 2021. FBI Crime Data Explorer.

[46] The Asian American Foundation Media Reporting. GitHub.

