# Supplementary material for: Comparing Media and Law Enforcement Reports on Anti-Asian Hate Incidents During the COVID-19 Pandemic: Data Visualization Approach
Source: JMIR Form Res. 2025 Sep 17;9:e70881. doi: 10.2196/70881 (PMC12489402; doi:10.2196/70881)
Supplement: Multimedia Appendix 1 [file formative_v9i1e70881_app1.docx]

Table S1. Categorization of incident types by data sources.

| Incident types | FBI | Media |
| --- | --- | --- |
| Assault | - Aggravated Assault - Aggravated Assault; Destruction/Damage/Vandalism of Property - Aggravated Assault; Intimidation - Aggravated Assault; Murder and Nonnegligent Manslaughter - Aggravated Assault; Purse-snatching; Weapon Law Violations - Simple Assault - Aggravated Assault; Simple Assault - Kidnapping/Abduction - Murder and Nonnegligent Manslaughter - Robbery - Rape | - Assault - Assault, Harassment - Assault, Harassment, Property-related - Assault, Harassment, Shunning - Assault, Property-related - Assault, Property-related, Harassment |
| Property-related | - All Other Larceny - All Other Larceny; Simple Assault - Arson - Burglary/Breaking & Entering - Burglary/Breaking & Entering; Destruction/Damage/Vandalism of Property - Burglary/Breaking & Entering; Destruction/Damage/Vandalism of Property; Intimidation - Burglary/Breaking & Entering; Intimidation - Destruction/Damage/Vandalism of Property - Destruction/Damage/Vandalism of Property; Intimidation - Destruction/Damage/Vandalism of Property; Intimidation; Simple Assault - Destruction/Damage/Vandalism of Property; Robbery - Destruction/Damage/Vandalism of Property; Simple Assault - Destruction/Damage/Vandalism of Property; Theft of Motor   Vehicle Parts or Accessories   - Destruction/Damage/Vandalism of Property; Weapon Law Violations - Destruction/Damage/Vandalism of Property; Motor Vehicle Theft - Motor Vehicle Theft - Theft from Motor Vehicle - Theft of Motor Vehicle Parts or Accessories - Shoplifting | - Property-related - Property-related, Assault - Property-related, Harassment |
| Harassment | - Extortion/Blackmail; Intimidation - Intimidation - Intimidation; Simple Assault | - Harassment - Harassment, Assault - Harassment, Assault, Property-related - Harassment, Property-related - Harassment, Shunning |
| Shunning | NA | - Shunning - Shunning, Harassment - Shunning, Harassment, Assault - Shunning, Harassment, Property-related |
| Other | - Drug/Narcotic Violations - False Pretenses/Swindle/Confidence Game; Simple Assault - Hacking/Computer Invasion - Not Specified - Weapon Law Violations - Counterfeiting/Forgery - False Pretenses/Swindle/Confidence Game - Identity Theft - Weapon Law Violations | NA |

Table S2. Categorization of incident locations by data sources.

| Incident locations | FBI | Media |
| --- | --- | --- |
| Transportation | - Air/Bus/Train Terminal | - Public area, Transportation - Transportation - Transportation, Business |
| Business | - Amusement Park - Arena/Stadium/Fairgrounds/Coliseum - Bank/Savings and Loan - Bar/Nightclub - Commercial/Office Building - Construction Site - Convenience Store - Department/Discount Store - Gambling Facility/Casino/Race Track - Grocery/Supermarket - Grocery/Supermarket; Highway/Road/Alley/Street/Sidewalk - Hotel/Motel/Etc. - Industrial Site - Liquor Store - Restaurant - Service/Gas Station - Shopping Mall - Specialty Store - Rental Storage Facility | - Business - Business, Public area |
| Public area | - Camp/Campground - Community Center - Dock/Wharf/Freight/Modal Terminal - Field/Woods - Government/Public Building - Government/Public Building; Other/Unknown - Highway/Road/Alley/Street/Sidewalk - Jail/Prison/Penitentiary/Corrections Facility - Lake/Waterway/Beach - Park/Playground - Parking/Drop Lot/Garage - Rest Area - Shelter-Mission/Homeless | - Public area |
| Place of worship | - Church/Synagogue/Temple/Mosque | - Place of worship |
| Online | - Cyberspace | - College or university, Online - Online - Online, Business - Online, College or university - Online, Home or housing - Online, Place of worship - Online, Unknown |
| Health care setting | - Daycare Facility - Drug Store/Doctor's Office/Hospital | - Hospital or clinic |
| Home or housing | - Parking/Drop Lot/Garage; Residence/Home - Residence/Home | - Home or housing |
| College or university | - School-College/University | - College or university |
| School | - School-Elementary/Secondary | - School |
| Other | - Other/Unknown | - Unknown |
